# Native and non-native language contexts differently modulate mood-driven electrodermal activity

**DOI:** 10.1038/s41598-022-27064-3

**Published:** 2022-12-26

**Authors:** Marcin Naranowicz, Katarzyna Jankowiak, Maciej Behnke

**Affiliations:** 1 Faculty of English, Adam Mickiewicz University, Poznań, Grunwaldzka 6, 60–780 Poznań, Poland; 2 Faculty of Psychology and Cognitive Sciences, Adam Mickiewicz University, Poznań, Poland

**Keywords:** Human behaviour, Emotion

## Abstract

Bilingual speakers have been consistently observed to experience reduced emotional sensitivity to their non-native (L2) relative to native (L1) language, particularly to the negatively-valenced L2 content. Yet, little is known about how the L1 and L2 contexts physiologically influence bilinguals’ affective states, such as moods. Here, we show that bilinguals may be less physiologically sensitive to mood changes in the L2 compared to the L1 context. Polish–English bilinguals operating in either the L1 or the L2 mode (elicited via reading L1 and L2 sentences) watched positive and negative mood-inducing films while their electrodermal activity was measured. We observed a greater number of skin conductance responses in the negative compared to positive mood condition in the L1 context only, indexing decreased sensitivity to mood changes in the L2 relative to the L1 mode in bilinguals. Also, skin conductance amplitudes were overall increased in the L2 compared to the L1 context, pointing to increased cognitive load when operating in L2. These findings together suggest that bilinguals experience decreased sensitivity to mood changes in their less dominant language due to L2 processing requiring greater cognitive engagement.

## Introduction

In today’s multilingual world, communicating affectively in the native (L1) and non-native (L2) language may pose personal and interpersonal challenges. This has been reflected in empirical evidence pointing to the emotional detachment in L2, which suggests that bilinguals process emotional content in a language-dependent manner (see Jończyk^1^ for a review). Importantly, though recent research has suggested that emotional responding both influences and is influenced by the language of operation (L1 vs. L2)^2,3^, the underlying mechanisms guiding the interplay between bilinguals’ affective states and L2 mood regulatory abilities remain largely under-researched. To address this research gap, the present study focuses on how being in an L1 and an L2 mode (i.e., processing information in L1 and L2) influences bilinguals’ physiological reactivity when induced experimentally into a positive and a negative mood. In contrast to emotion (i.e., a short-lived, specific, and full-blown affective state), mood is defined here as a slowly-changing, low-intensity background affective state that rather unobtrusively fluctuates over time from feeling positive (i.e., good and pleasant) to negative (i.e., bad and unpleasant)^4^.

### Affect and bilingualism

Accumulating evidence has demonstrated increased emotional distance when unbalanced bilinguals process emotional content (e.g., words, sentences, or narratives) in their less dominant language (see Caldwell–Harris^5^; Jończyk^1^ for reviews). Such attenuated emotional reactivity has been argued to be particularly salient for negatively-valenced L2 content, as supported by self-report^6^, behavioural^7^, physiological^8^, electrophysiological^9^, and neuroimaging^10^ research. For instance, Jończyk et al.^9^ observed decreased N400 (i.e., an event-related potential component marking lexico-semantic processing) amplitudes for L2 negative words compared to L1 negative words as well as L1 and L2 positive words. Such a pattern points to the less automatic lexico-semantic access to negative content in one’s less dominant language, as driven by a decreased activation of the emotional load encoded in negative words in L2. Recent evidence has also suggested that a positive and a negative mood may differently modulate cognitive mechanisms engaged in L1 and L2 processing in unbalanced bilinguals^3,11,12^. For instance, Naranowicz et al.^3^ observed that meaningful sentences evoked higher late positive complex (i.e., an event-related potential component marking semantic integration and re-analysis) amplitudes in L2 than in L1. Such a pattern suggests that a negative mood leads to the suppression of full semantic integration in L2 only, possibly as a result of high and accumulated cognitive demands triggered by both being in a negative mood and operating in a non-dominant language.

Similarly, previous studies on emotion regulation have pointed to language-dependent emotional reactivity^2,13^. For instance, Morawetz et al.^2^ observed more effective regulation of negative emotions elicited by aversive (i.e., negative) pictures in L2 than in L1 when performing a task distracting bilinguals from the experienced emotions. Such an L2 advantage has been hypothesised to result from an increased number of cognitive resources bilinguals need to allocate to processing their non-dominant language. This consequently limits the resources available for emotion recognition and analysis, thus attenuating bilinguals’ emotional responding^3^. Together, previous research has pointed to a complex relationship between unbalanced bilinguals’ current affective states, especially negative emotions and moods, and their language of operation (L1 vs. L2). First, bilingual speakers may be less sensitive to negative content when operating in their L2 compared to L1^1^. Second, being in a negative mood may impede semantic integration in L2 relative to L1^3^. Third, unbalanced bilinguals have been observed to regulate their negative emotions more when operating in L2 than in L1^2,13^.

### Electrodermal activity and emotion processing

Changes in affective states are inherently accompanied by changes in the autonomic nervous system, as may be reflected in skin conductance (SC) fluctuations^14^. SC is an example of electrodermal activity (EDA)—an umbrella term for all electrical activity observed in the skin^15,16^. From a physiological perspective, EDA measurements are used to acquire parameters that reflect sweat gland activity, as evident in changes in skin conductance^15^. Such fluctuations index changes in peripheral autonomic activity, as modulated by several different psychological processes, including attention, habituation, cognitive effort, and emotional arousal^17,18^. Consequently, EDA needs to be perceived as a multifaceted phenomenon that does not mark a single psychological process but rather reflects an array of intercorrelated psychophysiological mechanisms^19^.

From an evolutionary perspective, regulating body temperature, as evinced in the EDA changes, reflects the “fight-or-flight” response^20^. Increased EDA responses have also been strongly correlated with heightened anxiety, threat, and the processing of emotionally arousing stimuli^21^. For this reason, EDA is frequently treated as a marker of emotional arousal^22^. EDA is used to measure physiological responding when experiencing emotions, with increased EDA reactivity reflecting increased physiological arousal in response to experienced emotions^23^. Importantly, it has been observed that negative emotions are typically more arousing than positive emotions, thus eliciting more pronounced EDA^24–26^.

The most common SC parameters include skin conductance level (SCL) and skin conductance response (SCR). SCL indexes tonic activity, manifesting skin conductivity over longer time intervals and indicating a general, stimulus-non-specific, level of arousal^19^. SCR, on the other hand, is characterised by phasic activity (i.e., fast and short fluctuations in the EDA), as reflected in sharp amplitude peaks that are usually observed in response to an external stimulus and are modulated by the stimulus intensity^15,27,28^. Additionally, the mean amplitude of skin conductance during the occurrence of SCRs is reflected in skin conductance amplitude (SCA). Interestingly, though both SCR and SCL mark the activation of the autonomic nervous system, it has been suggested that they index different brain mechanisms^29,30^, which thus stresses the importance of computing both SCR and SCL measures to provide a more valid and thorough insight into the topic under investigation.

### Electrodermal activity and emotion processing in bilinguals

EDA measures, especially SCR, have also been employed to test how bilingual speakers respond in their respective languages to emotional content, including positive and negative words, sex and taboo words, insults, childhood reprimands, or endearments (see Kazanas et al.^31^ for a review). Consistent with the *emotional context of learning* hypothesis^5^, bilingual speakers who acquire their L2 in formal (i.e., instructional) contexts typically exhibit increased SCR^8,32^ and SCL^28,33^ patterns to emotionally laden compared to neutral content in their L1, with this difference being absent in L2. In contrast, bilinguals who acquired their L2 naturalistically (i.e., via social interactions) frequently show no between-language differences in their SCR to emotionally-laden linguistic content^34,35^.

Research attention has recently been directed to bilinguals’ physiological reactivity in different affective states as modulated by the language context. García-Palacios et al.^36^ tested how the language of the task (L1 vs. L2) influences bilinguals’ physiological responses (i.e., pupil dilations and SCRs) in a fear conditioning experiment. Fear conditioning is a psychopathological mechanism activated via a repetitive presentation of a neutral stimulus in the presence of a threat of receiving mild electric shocks. The results revealed larger pupil size and larger SCRs in the threat compared to neutral conditions. The effect was significantly reduced in L2 relative to L1, suggesting that processing information in the L2 context may reduce the intensity of the experienced fear or anxiety. Importantly, García-Palacios et al.^36^ also noted overall increased physiological reactivity in L2 than L1, as indexed by the pupillary responses, reflecting higher cognitive demands imposed by performing cognitive tasks in L2^37^.

Altogether, growing evidence showing decreased sensitivity to emotional (especially negative) L2 content (see Jończyk^1^ for a review) has recently been corroborated by research on mood^3^ and emotion regulation^2^, pointing to a reciprocal relationship between affective states and the language of operation. Moreover, emerging physiological evidence has suggested that the language context may actively modulate bilinguals’ individual affective states, such as anxiety^37^. Still, what remains an open question is whether the differential effects of L1 and L2 contexts on bilinguals’ physiological reactivity are limited to the emotions elicited while reading emotionally-laden language or are generalisable to bilinguals’ positive and negative moods—current background affective states.

### Research aims and hypotheses

The present study aimed to examine how the L1 and L2 context (i.e., processing information in L1 or L2) influences bilinguals’ physiological reactivity in a positive and a negative mood. To this end, proficient Polish–English bilinguals who acquired English (L2) in a formal context were experimentally induced into a positive and a negative mood via affectively evocative film clips, presented alternately with L1 and L2 semantically complex sentences, while their EDA was being measured. Half of the participants made semantic decisions on L1 sentences, and another half on L2 sentences, as a means to put them in the L1 or L2 mode, respectively. Crucially, similarly to García-Palacios et al.^36^, we measured bilingual speakers’ physiological reactivity while exposed to non-narrative mood-inducing films in an L1/L2 mode of information processing (i.e., *in-between* reading neutral L1 and L2 sentences), and not *while* reading emotionally—laden L1 and L2 content. This allowed us to further explore overall contextual L1 and L2 effects on physiological reactivity in bilinguals being in a given affective state (i.e., a positive and a negative mood), rather than physiological responses to L1 and L2 emotional content. We employed SCR as a primary measure of skin conductance in the present study due to its prevalent use in previous research on emotional reactivity in bilinguals[e.g., ^34^^,^^38^^,^^39^]. We additionally analysed SCA and SCL in an exploratory fashion to gain a better understanding of participants’ emotional states. Building upon previous research^2,3,28^, we expected to observe larger SCRs in the negative compared to positive mood condition in L1, with no between-mood differences in L2, which would point to the differential effects of the L1 and L2 contexts on bilinguals’ current affective states.

## Results

### Mood ratings

**Figure 1:**
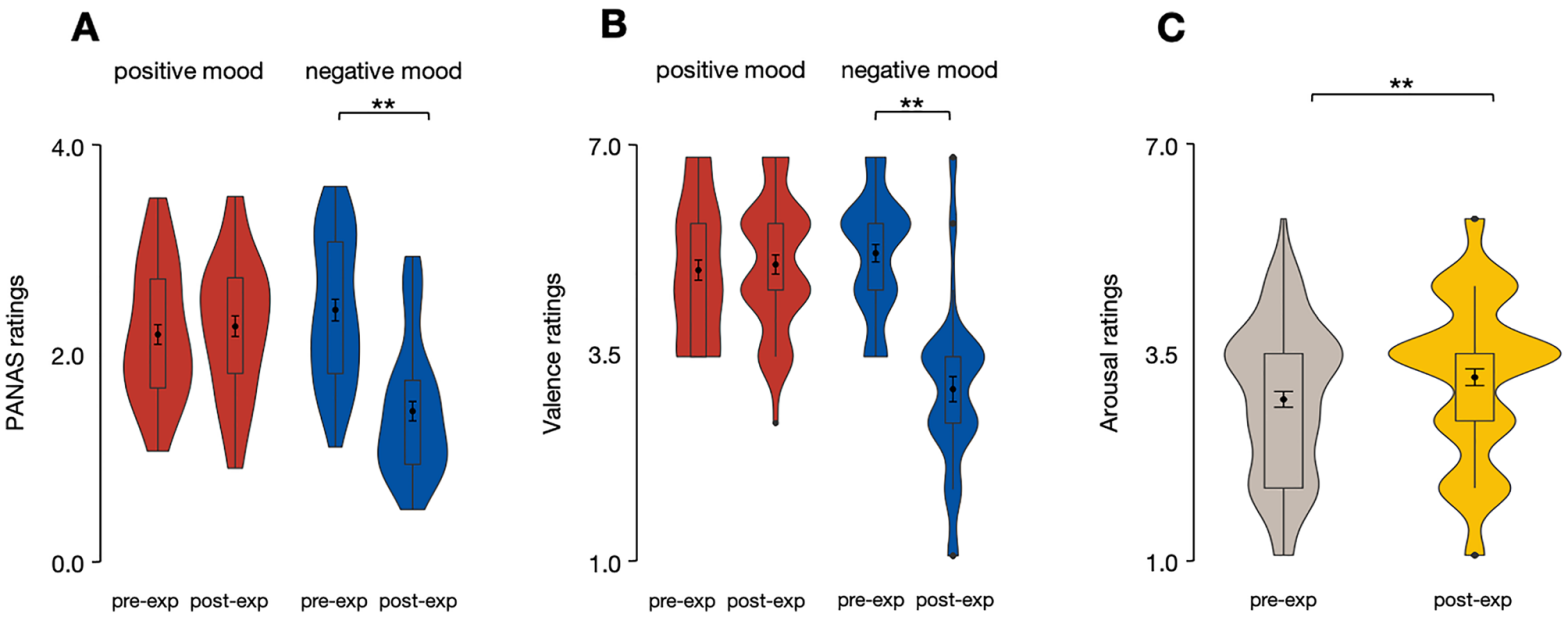
The PANAS (**A**), valence (**B**) and arousal (**C**) ratings pre- relative to post-experiment. Error bars represent 95% confidence intervals.

The analysis of the PANAS ratings showed no fixed effect of Language as well as no interactions between Language and other factors, *p* > 0.05. However, there were fixed effects of both Film type, *b* = 0.32, SE = 0.07, *t*(135) = 4.55, *p* < 0.001, and Testing time, *b* = 0.44, SE = 0.07, *t*(135) = 6.17, *p* < 0.001, along with a Film type × Testing time interaction, *b* = –1.14, SE = 0.14, *t*(135) = − 8.04, *p* < 0.001. As expected, planned comparisons showed a decrease in the PANAS ratings post- compared to pre-experiment in the negative mood condition (*M*_*Pre-experiment *_= 2.22, 95% CI[2.02, 2.42]; *M*_*Post-experiment*_ = 1.46, 95% CI[1.25, 1.66]), *b* = 1.00, SE = 0.10, *t*(135) = 10.10, *p* < 0.001, with no differences in the positive mood conditions (*M*_*Pre-experiment*_ = 2.47, 95% CI[2.26, 2.67]; *M*_*Post-experiment*_ = 2.35, 95% CI[2.14, 2.55]), *b* = − 0.13, SE = 0.10, *t*(135) = − 1.27, *p* = 1.00 (see Fig. 1A).

Similarly, the analysis of the valence ratings showed no fixed effect of Language as well as no interactions between Language and other factors, *p* > 0.05. Yet, there were fixed effects of both Film type, *b* = 0.82, SE = 0.12., *t*(135) = 7.05, *p* < 0.001, and Testing time, *b* = 0.97, SE = 0.12, *t*(135) = 8.39, *p* < 0.001, along with a Film type × Testing time interaction, *b* = –2.10, SE = 0.23, *t*(135) = –9.04, *p* < 0.001. As expected, planned comparisons showed a decrease in the valence ratings post- compared to pre-experiment in the negative mood condition (*M*_*Pre-experiment*_ = 5.53, 95% CI[5.23, 5.84]; *M*_*Post-experiment*_ = 3.50, 95% CI[3.19, 3.81]), *b* = 2.03, SE = 0.16, *t*(135) = 12.46, *p* < 0.001, with no differences in the positive mood conditions (*M*_*Pre-experiment*_ = 5.29, 95% CI[4.98, 5.60]; *M*_*Post-experiment*_ = 5.37, 95% CI[5.06, 5.68]), *b* = − 0.08, SE = 0.16, *t*(135) = − 0.49, *p* = 1.00 (see Fig. 1B). Therefore, both self-reported mood measures (i.e., the PANAS and valence ratings) confirmed that, irrespective of the language mode, participants experienced a decrease in their emotional state in the negative mood condition. They also maintained a comparably positive emotional state to the one they had before mood induction in the positive mood condition.

The analysis of the arousal ratings again showed no fixed effect of Language as well as no interactions between Language and other factors, *p* > 0.05. At the same time, the analysis revealed a fixed effect of Testing time, *b* = − 0.32, SE = 0.13, *t*(135) = − 2.50, *p* = 0.014, whereby participants reported feeling more physiologically aroused post- relative to pre-experiment (*M*_*Pre-experiment*_ = 3.33, 95% CI[3.04, 3.62]; *M*_*Post-experiment*_ = 3.66, 95% CI[3.37, 3.95]) (see Fig. 1C).

### Electrodermal activity

**Figure 2:**
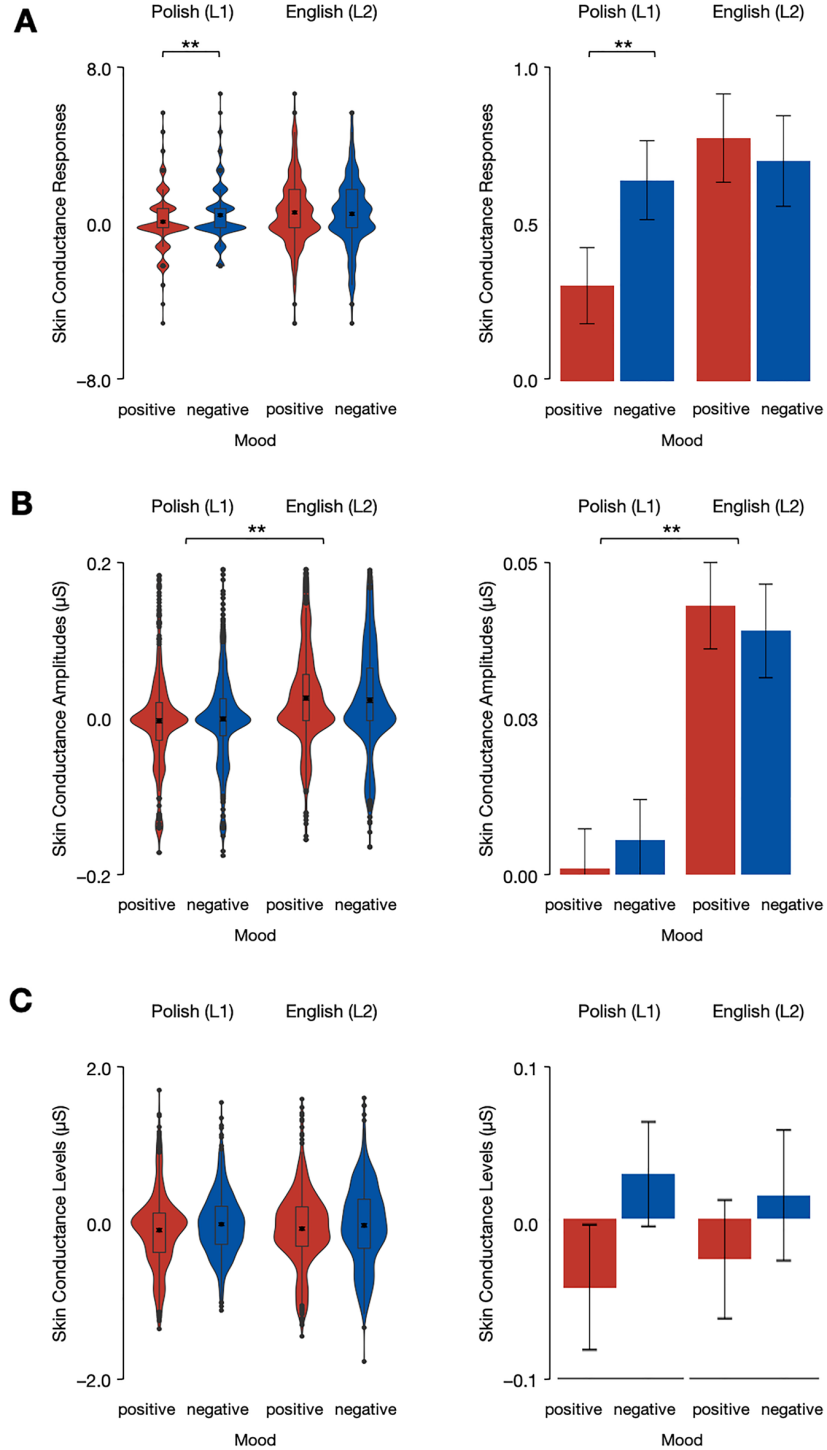
Skin conductance responses (**A**), amplitudes (**B**), and levels (**C**) in English (L2) and Polish (L1) in the negative (blue) and positive (red) mood conditions. Error bars represent 95% confidence intervals.

First, the analysis of skin conductance response (SCR; a marker of phasic electrodermal activity) showed a Mood × Language interaction, *b* =  − 0.59, SE = 0.23, *t*(43.3) =  − 2.51, *p* = 0.016. *Post-hoc* comparisons revealed a greater number of SCRs in the negative compared to the positive mood condition in the L1 (Polish) condition (*M*_*PositiveMood*_ = 0.28, 95% CI[− 0.23, 0.80]; *M*_*NegativeMood*_ = 0.75, 95% CI[0.24, 1.25]), *b* = − 0.46, SE = 0.18, *t*(50.8) =  − 2.58, *p* = 0.013, with no between-mood differences in L2 (English) condition (*M*_*PositiveMood*_ = 0.83, 95% CI[0.35, 1.31]; *M*_*NegativeMood*_ = 0.70, 95% CI[0.22, 1.17]), *b* = 0.13, SE = 0.17, *t*(52.7) = 0.77, *p* = 0.443. Then, the analysis of SCRs showed no fixed effects of Language, *b* =  − 0.25, SE = 0.32, *t*(44.8) =  − 0.77, *p* = 0.443, or Mood, *b* =  − 0.18, SE = 0.16, *t*(54.9) =  − 1.38, *p* = 0.174 (see Fig. 2A).

Second, the analysis of SCA (i.e., a measure of the SCR magnitude) revealed a fixed effect of Language, *b* =  − 0.03, SE = 0.01, *t*(44.7) =  − 2.77, *p* = 0.009, whereby SCAs were higher in the L2 (English) (*M* = 0.04, 95% CI[0.02, 0.06]) compared to L1 (Polish) condition (*M* = 0.001, 95% CI[− 0.01, 0.02]). Also, the analysis of the SCA data showed no fixed effect of Mood, *b* <  − 0.01, SE < 0.01, *t*(52.3) =  − 0.15, *p* = 0.882, as well as no Mood × Language interaction, *b* <  − 0.01, SE < 0.01, *t*(41.4) =  − 1.77, *p* = 0.084 (see Fig. 2B).

Third, the analysis of SCL (i.e., a marker of tonic electrodermal activity) revealed no fixed effects of Language, *b* =  − 0.01, SE = 0.10, *t*(48.3) =  − 0.11, *p* = 0.914, or Mood, *b* =  − 0.05, SE = 0.06, *t*(48.3) =  − 0.74, *p* = 0.466, as well as no Mood × Language interaction, *b* =  − 0.01, SE = 0.13, *t*(44.9) =  − 0.10, *p* = 0.923 (see Fig. 2C).

**Figure 3:**
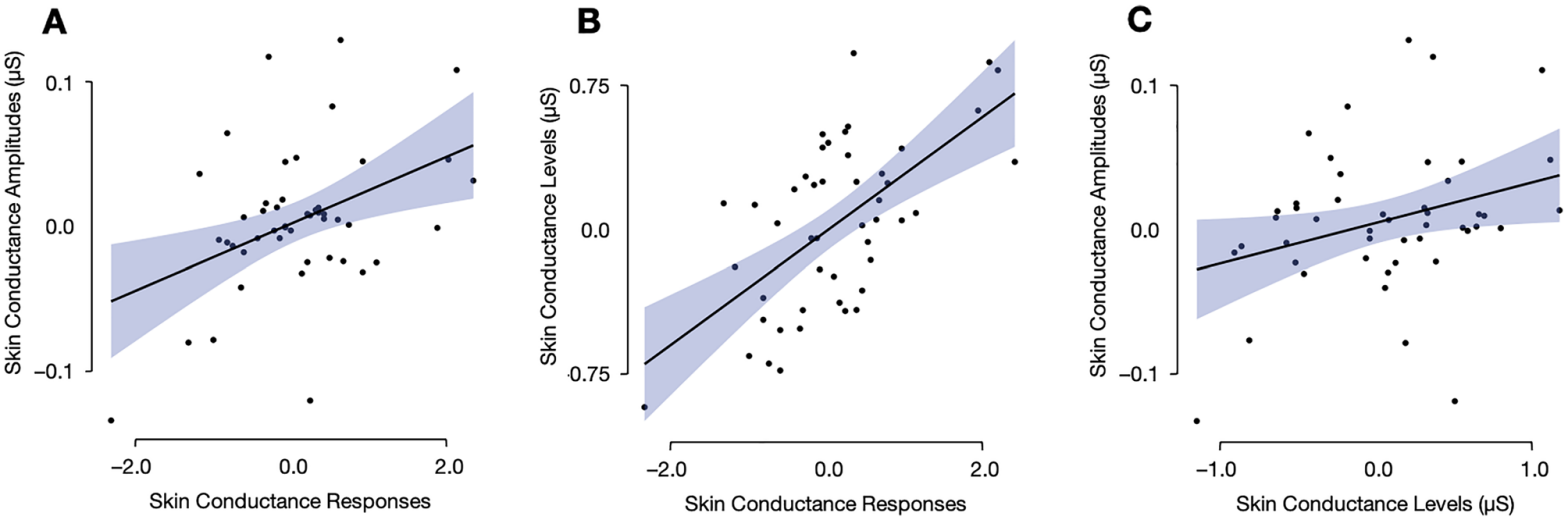
Correlation plots depicting the relationship between the skin conductance measures: SCA and SCR (**A**), SCL and SCR (**B**), as well as SCA and SCL (**C**).

To further explore the relationship between the individual EDA measures, we also calculated the difference in the EDA elicited in the positive and negative mood conditions for each respective measure. Correlational analyses pointed to positive associations among them all: SCA × SCR: *r* = 0.41, 95% CI[0.14, 0.63], *t*(45) = 3.05, *p* = 0.004 (see Fig. 3A); SCL × SCR: *r* = 0.62, 95% CI[0.40, 0.77], *t*(45) = 5.29, *p* < 0.001 (see Fig. 3B); SCA × SCL: *r* = 0.31, 95% CI[0.02, 0.55], *t*(45) = 2.17, *p* = 0.035 (see Fig. 3C).

## Discussion

The present study explored how the native (L1) and non-native (L2) language contexts (as elicited via reading L1 and L2 sentences of different semantic complexity) influence bilinguals’ physiological responses (i.e., SCRs, SCAs, and SCLs) in a positive and a negative mood (as evoked by affectively evocative films). Building on previous research on affect and bilingualism^2,3,28^, we expected increased physiological reactivity in a negative compared to a positive mood in the L1 context only.

Consistent with our hypothesis, we observed an increase in phasic physiological responding, as indexed by the SCR changes, in a negative compared to a positive mood in the L1 context only. This is consistent with previous research pointing to higher arousal levels reported when experiencing negative relative to positive affective states^25,33^, indicating a higher emotional intensity of negative stimuli^28^. In fact, in a recent meta-analysis, Joseph et al.^40^ estimated that negative content manipulated experimentally elicits almost two times stronger mood changes than positive content. Such increased affective resonance of negative content is in accordance with the *negativity bias* assumption^41,42^, pointing to negative emotions having stronger effects on attention, behaviour, decision-making, or affect than positive emotions due to their different adaptive functions (see Norris^43^ for a review). Specifically, negative, unlike positive, content is assumed to signal a potential threat to homeostatic balance, thereby eliciting survival-driven cognitive and physiological reactions^44^.

As predicted, we did not observe the modulations in SCRs by mood in the L2 mode. Such decreased physiological sensitivity to mood changes in L2 is consistent with previous studies showing increased emotional detachment when processing a foreign language acquired in formal (i.e., instructional) school settings^8,28,32,33^. Building upon such findings, Caldwell-Harris^5^ proposed the *emotional context of learning* hypothesis, suggesting that the lack of emotion-based communicative interactions (i.e., little immersion) when acquiring a language leads to lower emotional resonance due to the decreased connectivity between this language and emotion regulation systems. As indicated by the results of Language History Questionnaire^45^, our participants learnt English as a foreign language in instructional settings and used it mostly in non-emotional contexts. Consequently, decreased emotional resonance in L2 could result in decreased sensitivity to mood changes when operating in the L2 mode. This is also in accordance with the *foreign language effect* account^46^, associating the emotional detachment with a more global psychological distance in L2. Specifically, processing information in L2 has been observed to lead to less emotional and biased as well as more rational and utilitarian decision-making^47–49^.

Crucially, our findings also accord with those observed by García-Palacios et al.^36^, who explored the language context effects on bilinguals’ physiological responses in a fear-conditioning experiment. Similarly to our study, the authors observed that while bilingual speakers exhibited overall increased physiological responses (i.e., pupil dilation and SCRs) in the threat compared to the neutral condition, the intensity of such responses was significantly reduced when operating in L2 compared to L1. This suggests that a specific affective state, such as imminent threat-related anxiety or fear, may be less intensively experienced by bilinguals in the L2 than the L1 context. Altogether, our study, therefore, extends previous bilingual research on emotional language processing^8,28,32,33^ as well as individual affective states^36^, showing that a reduced L2 relative to L1 contextual effect on bilingual speakers’ phasic physiological reactivity may also be observed with relation to more global affective states, such as a positive or a negative mood.

We also observed more pronounced SCAs in the L2 compared to the L1 mode, irrespective of the mood type. Such a general effect of language context complements the mood–language interaction reflected in the SCR changes, indicating that the SCRs were overall more robust (i.e., had higher amplitudes) in the L2 than L1 mode. Increased physiological reactivity in the L2 compared to the L1 context has previously been linked with increased cognitive load invested when performing a cognitive task in the L2 relative to the L1 context^36,50–52^. In the present study, higher cognitive demands could have been reinforced by the task performed: bilinguals made semantic judgments on figurative language (i.e., novel metaphors along with literal and anomalous sentences), whose processing is more cognitively effortful in L2 relative to L1^53–55^. The comprehension of novel (i.e., unfamiliar) metaphors entails creative thinking and extended semantic analyses, and therefore more pronounced physiological responding in the L2 than L1 mode could result from more effortful lexico-semantic mechanisms engaged in L2 than L1 processing, and not necessarily from increased emotional arousal.

Moreover, a link between increased cognitive demands and overall increased physiological responding in L2 has been supported by recent research on emotional reactivity^36,52^. In addition to the interactive effect of the language context (L1 vs. L2) and the threat condition (threat vs. neutral) discussed above, García-Palacios et al.^36^ also observed overall larger pupillary responses in the L2 than L1 context, indicating that the L2-driven cognitive demands may lead to overall increased physiological reactivity during fear acquisition. Correspondingly, Caldwell–Haris and Ayçiçeği–Dinn^52^ observed more pronounced SCR patterns to ethical dilemmas in L2 relative to L1, potentially deriving from an increased cognitive load or stress associated with L2 processing. Interestingly, Caldwell–Haris and Ayçiçeği–Dinn^52^ proposed that such increased cognitive demands elicited by L2 may swamp or obscure emotional responding in L2.

Similar conclusions regarding the role of cognitive demands in L2 in emotional responding have recently been drawn from research on emotion regulation in the bilingual context (^2^; see however Vives et al.^56^ for recent neuroimaging evidence on affect labelling). Morawetz et al.^2^ observed that unbalanced bilinguals more effectively down-regulated negative emotions elicited via aversive pictures in L2 than L1 when performing a distraction task unrelated to emotional responding (i.e., implicit emotion regulation). Similarly to the results of research on physiological responding in bilinguals, such enhanced regulation of negative emotions in L2 compared to L1 was interpreted as resulting from increased cognitive efforts invested in L2 processing, thereby limiting the resources available for regulating negative emotions and attenuating emotional responding. Alternatively, more pronounced physiological reactivity in L2 relative to L1 may also be accounted for by *foreign language anxiety*^57,58^: a phenomenon observed during L2 usage, whereby bilinguals experience a feeling of apprehension when processing L2. Altogether, while the exact mechanisms driving such a foreign language effect remain unknown and require further research, we interpret the overall increased physiological responding to L2 relative to L1 in our study as reflecting greater cognitive resources allocated to processing semantically complex meanings in L2 and/or increased foreign language anxiety^57,58^, which might have obscured emotional responding in the L2 mode^2,52^.

Interestingly, even though we found strong correlations between all the three EDA parameters that we analysed (i.e., SCR, SCA, and SCL), the mood- or language-driven differences were reflected only in the phasic parameters: SCR and SCA, with no effects observed in SCL. It, therefore, seems that mood effects might exert a stronger influence on phasic rather than tonic skin conductance activity. In the same vein, the language context effects on physiological responding in a positive and a negative mood might be more evident in terms of fast and short fluctuations that are modulated by stimulus intensity. Such results seem to be in line with previous psychophysiological research indicating that, though both measures mark the activation of the autonomic nervous system, phasic and tonic parameters might index different brain mechanisms^29,30^. Further research is, nonetheless, needed to provide more thorough insights into how mood and the language of operation modulate different EDA indicators.

The present study carries some crucial practical implications for bilingual therapy. Indeed, much empirical evidence has pointed to the beneficial effects of bilingual patients both naturally and strategically code-switching between their respective languages in psychotherapy^59^, which might help them distance themselves from traumatic or shameful experiences^60,61^. Our study supports these findings, suggesting that bilinguals can more effectively down-regulate their current affective state in the L2 than L1 mode. Further research is still needed to disentangle the underlying mechanisms guiding the interplay between bilinguals’ affective states and L2 mood regulatory abilities in the context of the actual therapy-based experiences.

## Limitations

Importantly, the present study examined the interplay between mood and language contexts by studying female participants only, and thus our results might not be generalizable to the bilingual population in a broader sense. Our decision to include females only was dictated by early evidence pointing to a more robust role of mood in language processing in women than men^12,62^. This also corroborates prior research suggesting females’ greater sensitivity to emotions^63^, often reflected in more pronounced EDA patterns^64,65^. Future research should further explore the psychophysiological underpinnings of mood and language processing of both men and women, thus increasing the external validity of such investigations.

## Conclusion

The present study investigated how the native (L1) and non-native (L2) contexts modulate bilinguals’ physiological reactivity in two background affective states—a positive and a negative mood. Our results extend previous bilingual research on emotional language processing^8,28,32,33^ and individual affective states^36^ by showing that bilingual speakers may be less physiologically sensitive to mood fluctuations when operating in the L2 compared to the L1 context. This accords well with previous research on emotional detachment in L2 (see Jończyk^1^ for a review) and foreign language effects^46^. We also found an overall stronger magnitude of physiological responding in the L2 compared to the L1 mode, pointing to an overall increased cognitive load and/or stress when operating in L2 than in L1^2,36,52,58^. The present study also carries potential practical implications for bilingual psychotherapy.

## Methods

The data reported in the present paper were collected as a part of a larger experiment, investigating electrophysiological (EEG) responses to semantically complex meanings as modulated by mood (Jankowiak et al.^66^). The present paper reports only the aims, methodology, and results relevant to the EDA part of the study. Consequently, different participants were excluded from the analyses in the two papers.

### Participants

The original sample included 51 participants, 4 of whom were excluded from the analyses due to electrode detachment (*n* = 2) and an insufficient number of trials (*n* = 2) (see the *Data analysis* section for details). Consequently, the final sample included 47 Polish (L1) learners of English (L2), who were randomly allocated to either the L1 block (*n* = 22; *M*_*Age*_ = 24.45, 95% CI[22.56, 24.35], age range = 21–29) or the L2 block (*n* = 25; *M*_*Age*_ = 22.76, 95% CI[22.00, 23.52], age range = 21–30). A priori power analysis appropriate for linear mixed-effects models (LMMs)^67^ indicated that participation of 46 bilinguals would yield a power of 0.8 to detect medium-to-large effects (Cohen’s *d* > 0.50). All participants were female, given the previous studies pointing to gender-dependent mood effects on language processing^12,61^. Participants were students and graduates of English Studies at the Faculty of English, Adam Mickiewicz University, Poznań. Following de Groot^68^), they were categorised as highly proficient unbalanced late Polish–English bilinguals, who had acquired English (L2) in a formal learning context and had never lived in the L2 environment (see Table 1). Forty-four participants were right-handed (93.61%), two were left-handed (4.26%), and one was ambidextrous (2.13%). Participants had normal or corrected-to-normal vision and hearing and no neurological, mood, psychiatric, or language disorders. Moreover, the Big Five Inventory^69^, Empathy Quotient^70^, and Interpersonal Reactivity Index^71^ were used to assess participants’ personality and empathy-related traits, which could potentially interact with their susceptibility to mood manipulation (see Supplementary materials). For their participation, they received a gift card of 200 PLN and extra credit points.

**Table 1:** Participants’ sociolinguistic data (means with 95% CI).

	Polish (L1)	English (L2)
Proficiency^a^	n/a	86.68[84.88, 88.47]
Proficiency^b^	97.37[95.95, 98.73]	87.77[85.95, 89.58]
Dominance^b^	60.68[58.37, 62.99]	53.70[51.58, 55.82]
Immersion^b^	78.06[74.96, 81.17]	69.68[67.42, 71.94]
Listening skills^b^	6.96[6.90, 7.02]	6.26[6.09, 6.42]
Speaking skills^b^	6.74[6.61, 6.88]	5.96[5.78, 6.13]
Reading skills^b^	6.89[6.79, 7.00]	6.36[6.21, 6.51]
Writing skills^b^	6.68[6.48, 6.88]	5.98[5.78, 6.18]
Age of acquisition^b^	n/a	6.34[5.84, 6.84]

^a^LexTALE^72^ (percentages); ^b^Language History Questionnaire 3.0^45^ (as translated into Polish by Naranowicz & Witczak): the proficiency, dominance, and immersion scores (percentages); listening, speaking, reading, and writing skills (1—very low proficiency, 7—very high proficiency); age of acquisition (years).

### Materials

#### Mood-inducing stimuli

To experimentally evoke a positive and a negative mood, we adopted 28 highly arousing non-narrative affectively evocative animated film clips from Naranowicz et al.^12^. The film clips were rated in a norming study, which demonstrated that while the positive mood-inducing films (*n* = 14) were rated significantly higher on the valence scale than the negative mood-inducing ones (*n* = 14), both film types scored similarly on the arousal scale (see Naranowicz et al.^12^ for details). To sustain the evoked targeted mood, each selected film clip was additionally divided into two 45-s clips, which resulted in the presentation of 56 films (i.e., 42 min in total) in both mood conditions.

#### Linguistic stimuli

To put our participants into either the L1 or the L2 mode of processing^68^, we additionally used linguistic stimuli presented in either their L1 and L2. The stimuli were all adopted from a database by Jankowiak^73^, and included 180 Polish (L1) and 180 English (L1) sentences that were divided into three categories: 60 novel metaphors (e.g., *My heart is a drawer for secret feelings.*), 60 literal sentences (e.g., *This piece of furniture is a drawer filled with socks.*), and 60 anomalous sentences (e.g., *A bug is a drawer shut with a bang.*). Consequently, the sentences were of different semantic complexity, thus ensuring that participants actively engaged in their processing.

### Procedure

The experiment was carried out in the Neuroscience of Language Laboratory (Faculty of English, Adam Mickiewicz University, Poznań), located in the Center for Advanced Technology at Adam Mickiewicz University, Poznań. Participants were randomly assigned to one of the two language blocks: Polish (L1) or English (L2) (a counterbalanced order). They were seated in a dimly lit and quiet booth, 75 cm away from a LED monitor with a screen resolution of 1280 × 1024 pixels.

The mood-inducing and linguistic stimuli were presented with E-Prime 3.0 (Psychology Software Tools Inc.). The positive and negative mood-inducing film clips were presented in separate mood blocks. Participants watched 28 mood-inducing films in each such block (*n*_*Total*_ = 56) and were instructed to put themselves in the targeted mood^74^ by imagining themselves as one of the protagonists and sympathizing with them^75^. In between the mood blocks, participants watched a 10-min low-arousing non-narrative nature documentary, aiming at neutralizing the mood state induced by the first mood block and establishing the baseline for the physiological parameters^76^. To measure the effectiveness of the mood manipulation, participants were asked to rate their current affective state before and after each mood block on valence and arousal scales and to complete the Polish version of the PANAS^77^ (as translated into Polish by Fajkowska & Marszał-Wiśniewska^78^).

**Figure 4:**
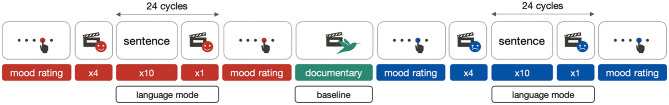
The experimental procedure following the assignment to the native (L1) or non-native (L2) language condition (“ × 4”, “ × 10”, and “ × 1” specify how many items were presented in a row).

In each mood block, participants first watched four film clips to induce the targeted mood state, and they were then alternately presented with another film clip to sustain the evoked state (i.e., continuous mood induction) and a set of 10 sentences in the language assigned to them (i.e., Polish or English). Participants were asked to perform a semantic decision task, wherein they decided if the presented sentences were meaningful or meaningless by pressing designated keys. Each mood block comprised 60 novel metaphoric, 60 literal, 60 anomalous, and 60 filler sentences (*n*_*Total*_ = 480). Asking participants to perform meaningfulness judgments aimed at activating meaning conceptualization mechanisms in the assigned language (i.e., Polish or English), thus putting participants in the target language mode^36^ when watching the mood-inducing film clips (see Fig. 4). The order of the mood blocks, key designation as well as the presented films and sentences were randomised and counterbalanced across participants (see Jankowiak et al.^66^, for details).

### Measures

#### Mood ratings

Participants rated their current affective state four times (i.e., before and after each of the two mood blocks) on 7-point mood valence (*1—highly negative, 7—highly positive*) and arousal (*1—highly unaroused, 7—highly aroused*) scales (i.e., bipolar dimensions). The scales were presented along with their corresponding 7-point Self-Assessment Manikin scales^79^. Each time, participants also completed the PANAS questionnaire^77^, including 10 positive mood-related adjectives (i.e., the Positive Affect scale) and 10 negative mood-adjective (i.e., the Negative Affect scale) (i.e., unipolar dimensions). Each of the twenty adjectives was rated on a 5-point scale (*1—very slightly or not at all, 5—extremely*). Participants’ ratings for individual positive and negative mood-related PANAS items were first summed to calculate the Positive and Negative affect scores. To make the PANAS results comparable with the valence ratings, we then analysed the ratio of the Positive to Negative affect scores.

#### Electrodermal activity

The EDA data were measured with Recorder 1.23 (Brain Vision Solutions Inc.), using the actiCHamp Plus amplifier (Brain Vision Solutions Inc.), sampled at 500 Hz and measured in microsiemens (μS). We used the electrodes with a contact area of 8 mm diameter, filled with the EDALYT Isotonic Electrolyte Gel, and attached with adhesive collars to the medial phalanges of digits II and IV of the non-dominant hand. Participants were asked to try not to move their hand during the experiment.

Next, the EDA signal was resampled to 150 Hz for the analysis. We used LabChart Pro 8.1 (ADInstruments Inc.) for calculating three measures of EDA: skin conductance responses (SCR), skin conductance amplitude (SCA), and skin conductance level (SCL) (see Behnke et al.^18^ for a review). SCL was calculated as the mean level of tonic skin conductance. SCR was determined from the SCL signal as transient increases of 0.01 μS over a time interval of measurement. SCA was determined as the peak value of the change in electrical conductance of the skin observed during the occurrence of SCR. SCR, SCA, and SCL were calculated for presentations of mood-inducing films and the neutralising nature documentary. Following Martínez–Rodrigo et al.^76^, a 45 s fragment of the neutralising nature documentary extracted from the middle of it was used for each participant as a baseline of the physiological parameters. The corresponding baseline values for each participant were then subtracted from their SCR, SCA, and SCL values and served as the reactivity scores for the analyses.

The SCR, SCA, and SCL data falling outside the value of the 2.5 interquartile range (IQR) were discarded from further analyses. Two participants were left with only 2% and 28% of the data and, consequently, were excluded completely from further analysis. Altogether, 11,72% of all data was discarded from the analysis.

### Data analysis

All statistical analyses were performed in R Core Team (2020).

#### Mood ratings

To ensure the effectiveness of our mood manipulation, we compared the valence, arousal, and PANAS (i.e., the ratio of the Positive to Negative affect scores) ratings pre- relative to post-experiment separately in each mood condition as planned comparisons, predicting increased/comparable mood ratings in the positive mood condition along with decreased mood ratings in the negative mood condition^3,12^. The mood rating data analysis conformed to a 2 (Time of testing: Pre-experiment vs. Post- experiment) × 2 (Film type: Positive vs. Negative) within-subject design, with Language (Polish[L1] vs. English[L2]) as a between-subject factor.

All three mood rating measures were analysed with linear mixed-effects models (LMM)^80–83^, using the *lme4* package^79^. Sum contrasts were applied to all categorical factors. A maximal model was first computed with a full random-effect structure, including subject- and item-related variance components for intercepts and by-subject and by-item random slopes for fixed effects^82^. When the data did not support the execution of the maximal model random structure, we reduced the model complexity to arrive at a parsimonious model^84^. To do so, we computed principal component analyses of the random structure and then kept the number of principal components that cumulatively accounted for 100% of the variance. *b* estimates and significance of fixed effects and interactions (*p*-values) were based on the Satterthwaite approximation for LMM (the *lmerTest* package)^85^. *Post-hoc* analyses were calculated using the *emmeans* package^86^.

#### Electrodermal activity

Similar to the mood rating data, SCR, SCA, and SCL reactivity were analysed with LMM (see *Mood ratings* for details), with a Mood (Positive vs. Negative) as a within-subject factor and Language (Polish[L1] vs. English[L2]) as a between-subject factor. The data for the first four film clips in each mood condition were not analysed, as these film clips were presented before the first set of sentences (i.e., participants were not yet put in the target language mode).

### Ethical approval

The study was conducted in accordance with the Declaration of Helsinki and approved by the Ethics Committee for Research Involving Human Participants at Adam Mickiewicz University, Poznań (Resolution No. 1/2020/2021, approved on 8 February 2021).

### Informed consent

Informed consent was obtained from all participants involved in the study.

## Supplementary Information

Supplementary Information.

## Data Availability

The raw data collected and analysed for the purpose of the current study are available at https://osf.io/s2exr/.
