# Supplementary material for: Native and non-native language contexts differently modulate mood-driven electrodermal activity
Source: Sci Rep. 2022 Dec 26;12:22361. doi: 10.1038/s41598-022-27064-3 (PMC9792545; doi:10.1038/s41598-022-27064-3)
Supplement: Supplementary file 1 — Supplementary Information. [file 41598_2022_27064_MOESM1_ESM.pdf]

Supplementary materials: Background questionnaires

Measures

Table 1. Participants' characteristics (mean percentages with 95% CI).

Handedness[1]	83.85 [74.33, 93.37]	Agreeableness[5]	86.17 [83.58, 88.76]
Positive affect[2]	63.01 [59.68, 66.34]	Conscientiousness[5]	73.87 [70.08, 77.66]
Negative affect[2]	41.43 [38.13, 44.73]	Emotional stability[5]	59.28 [55.23, 63.32]
Depression[3]	9.77 [6.99, 12.57]	Openness to experience[5]	62.09 [58.08, 66.09]
Anxiety[3]	8.94 [6.35, 11.54]	Perspective-taking[6]	40.27 [37.14, 43.41]
Stress[3]	6.64 [4.10, 9.17]	Fantasy scale[6]	50.38 [48.21, 52.55]
Empathy[4]	74.57 [68.48, 80.67]	Empathetic concern[6]	66.11 [62.60, 69.62]
Extraversion[5]	63.06 [57.90, 68.23]	Personal distress[6]	66.95 [63.39, 70.50]

Note. All values are presented as percentages. [1]Handedness Questionnaire [1] (HQ): left-handedness (100–28), ambidexterity (29–48), right-handedness (48–100); [2]Positive and Negative Affect Schedule [2] (PANAS; as translated into Polish by Fajkowska & Marszał-Wiśniewska [3]): Positive affect (interested, excited, strong, enthusiastic, proud, alert, inspired, determined, attentive, and active), Negative affect (distressed, upset, guilty, scared, hostile, irritable, ashamed, nervous, jittery, and afraid); [3]DASS-21 [4] (as translated into Polish by Makara-Studzińska et al.): normal (0–21%), mild (22–31%), moderate (32–48%), severe (49–64%), and extremely severe (65–100%) levels of depression, anxiety, and stress; [4]Empathy Quotient (EQ [5], as translated into Polish by Wainaina-Woźna): low (0–39%), average (40–64%), above average (65–78%), and high (79–100%) levels of empathy; [5]Big Five Inventory [59] (BFI; as translated into Polish by Strus et al., [6]): extraversion (talkativeness, activity, assertiveness vs. silence, passivity, reserve), agreeableness (kindness, trust, warmth vs. hostility, selfishness, distrust), conscientiousness (organisation, thoroughness, reliability vs. carelessness, negligence, unreliability), neuroticism (nervousness, moodiness, temperamentality vs. confidence, resilience), and openness to experience (imagination, curiosity, creativity vs. shallowness, imperceptiveness); [6]Interpersonal Reactivity Index (IRI [7], as translated into Polish by Kaźmierczak et al. [8]): perspective-taking scale ("the tendency to spontaneously adopt the psychological point of view of others"), fantasy scale (one's "tendencies to transpose themselves imaginatively into the feelings and actions of fictitious characters in books, movies, and plays"), empathetic concern scale ("other-oriented feelings of sympathy and concern for unfortunate others"), and personal distress scale ("self-oriented feelings of personal anxiety and unease in tense interpersonal settings").

Data analysis

Pearson correlation coefficients (r) were calculated to further explore whether there were any linear relationships between participants' individual characteristics and their physiological responses and mood ratings.

Results

The correlational analyses indicated that the IRI empathetic concern scores correlated negatively with the negative mood effect (i.e., the difference in valence ratings pre- relative to post-experiment in the negative mood condition) in the L1 condition, r = –.55, 95% CI [–.88, –.16], $t(20)$ = –2.93, p = .008 (see **Fig. 1A**). Also, the positive mood effect (i.e., the difference in valence ratings pre- relative to post-experiment in the positive mood condition) correlated positively with participants' BFI extraversion scores, r = .36, 95% CI [.08, .59], $t(45)$ = 2.61, p = .012 (see **Fig. 1B**), and the BFI emotional stability scores, r = .33, 95% CI [.04, .56], $t(45)$ = 2.31, p = .026 (see **Fig. 1C**).

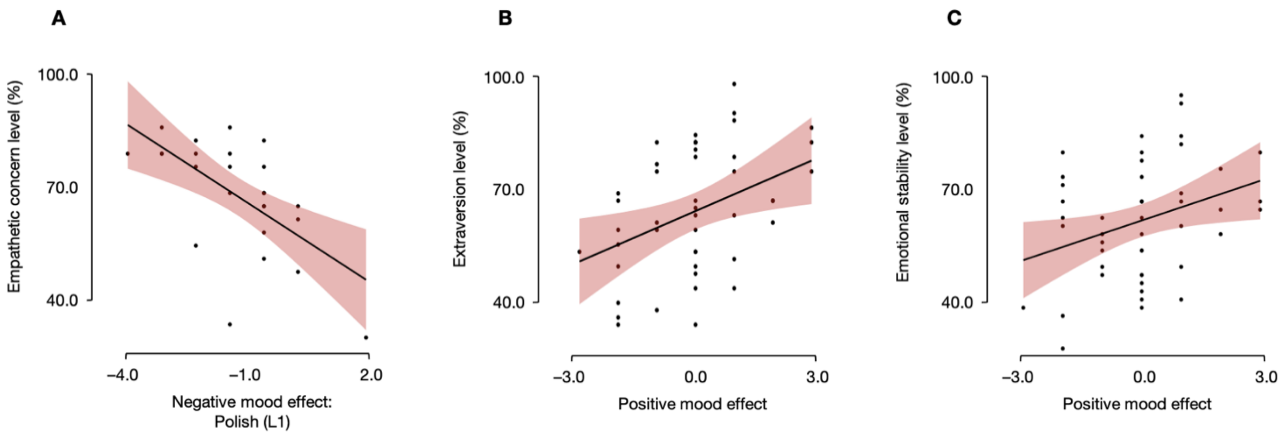

Fig. 1. Correlation plots depicting the relationship between the empathetic concern level and the negative mood effect in L1 (A), the extraversion level and the positive mood effect (B) as well as the emotional stability level and the positive mood effect (C).

Discussion

Our findings point to a potential linear relationship between bilinguals' empathy level and their emotional responding in the L1 context. Specifically, we found that bilinguals' responsiveness to negative mood induction, as indexed by changes in self-reported mood ratings, may increase proportionally to their empathy level, yet only in the L1 context. The presence of such an empathy effect in L1 in the negative mood condition most likely reflects the empathy-evoking nature of the negative mood-inducing films used in our study. Namely, the negative, unlike positive, films depicted fictional characters suffering (e.g., experiencing a loss of a beloved person, being bullied, witnessing domestic violence, etc.), which could consequently elicit a growingly intense empathetic response and an increase in a negative mood in those particularly empathetic ones. Crucially, the absence of a comparable mood–empathy relationship in the L2 context further evinces that our bilingual participants could not experience comparably strong negative emotions in L2 as in L1 [9]. Further research should therefore concentrate of a potentially mediating role of empathy in emotional responding in L1 and L2, especially given that early evidence has suggested that the L2 context promotes pro-social behaviours and the empathy bilingual speakers experience towards others [10–11].

Our findings also suggest that the responsiveness to the positive mood manipulation may be linked to participants' personality traits, such as extraversion. This is consistent with previous evidence showing that increased extraversion can be linked to higher reactivity to positive stimuli [12], including increased responsiveness to positive mood manipulation [13–14]. Neuroimaging research has partially accounted for this finding, consistently demonstrating increased brain activity in extraverts relative to introverts in the brain regions such as amygdala or striatum, both particularly sensitive to positivity-driven reward and approach behaviour (see DeYoung & Gray [15] for a review). Our results thus confirm that more extraverted bilinguals may report increased emotional reactivity when exposed to positive mood induction, irrespective of the language context.

References

1. Oldfield, R. C. The assessment and analysis of handedness: The Edinburgh inventory. *Neuropsychologia* **9**, 97–113 (1971).
2. Watson, D., Clark, L. A. & Tellegen, A. Development and validation of brief measures of positive and negative affect: The PANAS scales. *Journal of Personality and Social Psychology* **54**, 1063–1070 (1988).
3. Fajkowska, M. & Marszał-Wiśniewska, M. Właściwości psychometryczne Skali Pozytywnego i Negatywnego Afektu-Wersja Rozszerzona (PANAS-X). Wstępne wyniki badań w Polskiej próbie. [Psychometric properties of the Positive and Negative Affect Schedule-Expanded Form (PANAS-X). The study on a Polish sample.]. *Przegląd Psychologiczny* **52**, 355–387 (2009).
4. Lovibond, P. F. & Lovibond, S. H. The structure of negative emotional states: Comparison of the Depression Anxiety Stress Scales (DASS) with the Beck Depression and Anxiety Inventories. *Behaviour Research and Therapy* **33**, 335–343 (1995).
5. Baron-Cohen, S. & Wheelwright, S. The Empathy Quotient: An Investigation of Adults with Asperger Syndrome or High Functioning Autism, and Normal Sex Differences. *J Autism Dev Disord* **34**, 163–175 (2004).
6. Strus, W., Cieciuch, J. & Rowiński, T. The Circumplex of Personality Metatraits: A Synthesizing Model of Personality Based on the Big Five. *Review of General Psychology* **18**, 273–286 (2014).
7. Davis, M. A Multidimensional Approach to Individual Differences in Empathy. *JSAS Catalog Sel. Doc. Psychol.* **10**, (1980).
8. Kaźmierczak, M., Plopa, M. & Retowski, S. Skala Wrażliwości Empatycznej Sylwiusz Retowski. **50**, 9–24 (2007).
9. Liu, C., Wang, H., Timmer, K. & Jiao, L. The foreign language effect on altruistic decision making: Insights from the framing effect. *Bilingualism* 1–9 (2022) doi:10.1017/S1366728922000128.
10. Dewaele, J.-M. & Wei, L. Multilingualism, empathy and multicompetence. *International Journal of Multilingualism* **9**, 352–366 (2012).
11. Wu, Y. J., Liu, Y., Yao, M., Li, X. & Peng, W. Language contexts modulate instant empathic responses to others' pain. *Psychophysiology* **57**, e13562 (2020).
12. Gross, J. J., Sutton, S. K. & Ketelaar, T. Relations between affect and personality: Support for the affect-level and affective reactivity views. *Personality and Social Psychology Bulletin* **24**, 279–288 (1998).
13. Stafford, L. D., Ng, W., Moore, R. A. & Bard, K. A. Bolder, happier, smarter: The role of extraversion in positive mood and cognition. *Personality and Individual Differences* **48**, 827–832 (2010).
14. Vuoskoski, J. K. & Eerola, T. The role of mood and personality in the perception of emotions represented by music. *Cortex* **47**, 1099–1106 (2011).
15. DeYoung, C. G. & Gray, J. R. Personality neuroscience: Explaining individual differences in affect, behaviour and cognition. in *The Cambridge handbook of personality psychology* 323–346 (Cambridge University Press, 2009). doi:10.1017/CBO9780511596544.023.
